# Supplementary material for: The resolution of face perception varies systematically across the visual field
Source: PLoS One. 2024 May 13;19(5):e0303400. doi: 10.1371/journal.pone.0303400 (PMC11090322; doi:10.1371/journal.pone.0303400)
Supplement: Attachment — Submitted filename: Response to Reviews.docx [file pone.0303400.s001.docx]

***Response to Reviews: Morsi et al, The resolution of face perception varies systematically across the visual field***

Firstly, we wish to thank the editor and reviewer for their comments on our manuscript. We have made a number of changes to improve both the clarity in the description of our methods, and the explanations of what our results may suggest. Below, reviewer comments are in blue and our responses are in black. In the revised manuscript, new sections have been highlighted with tracked changes and comments.

***Reviewer 1***

*The conclusion of the paper seems to be that face perception per se does not vary systematically across the visual field - performance only changes because of low-level differences in contrast sensitivity. This conclusion is not well encapsulated by the current title.*

Our aim with the title is to refer to face perception more generally, broadly emphasising that our *ability to resolve faces varies around the visual field systematically.* This refers to our finding that acuity thresholds for both upright and inverted faces show the characteristic horizontal-vertical and upper-lower visual field anisotropies, and contrasts this result with the prediction from prior work that face perception might vary with e.g. completely idiosyncratic patterns across individuals. Our findings then naturally raise the question of the origin of these variations. While we agree that variations in low-level factors may indeed contribute to the anisotropies measured for faces (e.g. the radial bias, as discussed on p16, lines 402-412), the presence of large inversion effects demonstrates that face-specific processes were engaged by our gender recognition task. If performance on our task were solely due to low-level limitations such as contrast sensitivity, we would not observe such a strong inversion effect.

Although the size of this inversion effect did not vary significantly around the visual field, as one might expect if face-selective processes were themselves to vary, we nonetheless observed anisotropies for the recognition of *both* upright and inverted faces. In other words, face-recognition performance varies around the visual field (as referred to in the title), but it is less clear whether the ‘special’ configural/holistic aspects of face perception vary in addition to this (at least as they contribute to the resolution of face perception). We interpret this pattern of results as suggesting firstly that face perception requires sampling across large regions of the visual field (larger than e.g. letter acuity, as the reviewer notes below). This sampling has greater efficiency for upright faces than inverted (given that inverted faces required larger stimulus sizes to achieve the same accuracy level), with variations in sampling for both processes around the visual field. However, the spatial pooling required to extract holistic and/or configural information does not however appear to vary across the visual field in addition to this baseline pattern of variations.

Relevant to the reviewer’s comment on acuity below, a similar argument can be made for variations in low-level visual acuity, which are limited by retinal (photoreceptor density) as well as neural factors (V1 receptive field size). We similarly suggest that face acuity likely involves limits on resolution within lower (e.g. the radial bias) as well as later stages of visual processing (e.g. neural properties of face-selective regions), which both contribute to variations in face perception. As additional work is needed to ascertain the exact origin of these anisotropies, we would prefer to leave the title as it is – referring to the variations in thresholds that we observe. To clarify these points, we have more strongly emphasised the importance of the observed inversion effects in this regard, both in the introduction to set up the experiments (pp4-5, lines 104-110), in the results (e.g. Experiment 1: p9, lines 229-232) and in the discussion (pp15-16). We have also clarified the overall interpretation of our results and the potential low- and high-level factors that may contribute to this with an additional paragraph in the discussion (pp16-17, lines 437-458).

*The whole study is based on only 8 male and 8 female faces that were viewed at different sizes for many hundreds of trials by each participant. There is a high probability that participants could have learned the faces based on a small number of conspicuous features and based their response on those. This is especially problematic given that there was audio feedback for correct/incorrect responses. If so, the visibility of those features would depend on contrast sensitivity rather than facial configuration, and this would explain the results.*

We were also concerned that the use of a relatively small number of faces for the gender judgement could have introduced biases towards individual faces and/or features. To minimise the presence of any particularly distinctive features within our face sets (both at the lowest level in terms of local variations in contrast and in terms of distinctive facial features like noses etc), we ran pilot tests to ensure that all faces were recognised with broadly similar accuracy levels (p6, lines 133-135). More importantly, if participants categorised face gender based solely on certain conspicuous face features, we should not see inversion effects. The fact that our 3 experiments consistently produced clear inversion effects suggests that we were indeed able to engage the use of face-selective processes with our paradigm, and that these judgements certainly cannot be solely reduced to the reliance on conspicuous low-level features through e.g. differences in contrast sensitivity or distinctive facial features. We have highlighted the importance of the inversion effects in the introduction (pp4-5, lines 104-110), and the presence of these inversion effects in the results (p15-16, lines 391-401) to make this point more clearly.

*Face gender was determined in a preliminary study to ensure >8/10 ratings. Were those trials completed with oval-windowed grayscale faces?*

The preliminary study where gender ratings were recorded did indeed involve greyscale faces in an oval aperture – this detail has been added on p5, line 123.

*Line 170 The maximum allowable lapse rate of either 0.05 or 0.1 was applied to improve curve fitting. How was the improvement in curve fitting defined and what criterion was selected for an acceptable fit?*

These lapses relate to errors made by participants at large face sizes (where performance should otherwise be at ceiling). For psychometric function fitting, the lapse rate was 0 as standard. Some participants produced sufficient lapse errors that the resulting functions clearly diverged from the data points upon visual inspection. In these cases, the maximum allowable lapse rate was increased to either 0.05 or 0.1 in order to improve curve fitting. This was done on an individual basis, starting with 0.05 and increasing to 0.1 if the fits still did not improve. 7 participants had an adjusted lapse rate in Experiment 1, 3 in Experiment 2, and none in Experiment 3. Importantly, the adjusted lapse rate was applied across all conditions for that participant, meaning that neither the inversion effect nor the anisotropies in performance can be attributed to this factor. Clarification on this process has been added to the manuscript on page 7, lines 173-179.

*The studies were repeated at a single eccentricity (10 degrees). Letter acuity at 10 degrees is approximately 0.43 degrees (Anstis, 1974), so face thresholds are around 6-8 times larger than letter acuities. It would be beneficial for the author’s argument to report if face size acuity scales as a constant ratio of letter acuity with eccentricity.*

We agree that investigating how face acuity thresholds vary over eccentricity would be interesting. In fact, we are currently conducting a series of experiments to examine this issue. The picture here is complicated by demonstrations that acuity is not the sole limitation on peripheral vision – rather, one must also consider crowding effects (particularly with complex stimuli such as faces) in order to understand our peripheral recognition abilities. Examining this in the current set of experiments would add a considerable number of conditions/experiments, significantly increasing the length and complexity of the manuscript. For clarity of the results and to ensure that polar angle variations were the main focus of the paper, we have chosen to explore eccentricity variations and their correlates in a subsequent study. Nonetheless, as the reviewer highlights, it is indeed the case that our (upright) face acuity thresholds are approximately 8-13 times larger than that of letter acuity at 10° eccentricity. We agree that this is an important link to make, and we now make mention of this relationship in the discussion on p17, lines 433-437.

Finally, we have removed information about funding from the Acknowledgements section of the manuscript and confirm that our funding statement should read: “This work was supported by the Biotechnology and Biological Sciences Research Council [grant number BB/J014567/1]. The funders had no role in study design, data collection and analysis, decision to publish, or preparation of the manuscript.”.
